# Oncofertility care in young women and the outcomes of pregnancy over the last 5 years

**DOI:** 10.2144/fsoa-2020-0169

**Published:** 2021-02-02

**Authors:** Sayuri Takahashi, Akihito Horie, Sachi Yamamura, Akeo Kawamura, Ayaka Yamaguchi, Masumi Sunada, Hirohiko Tani, Haruta Mogami, Junzo Hamanishi, Eiji Kondoh, Masaki Mandai

**Affiliations:** 1 Department of Gynecology & Obstetrics, Kyoto University, 54 Shogoin Kawahara-cho, Sakyo-ku, Kyoto 6068507, Japan

**Keywords:** cancer survivors, cryopreservation of oocytes, cryopreservation of ovarian tissues, GnRH agonist, oncofertility care

## Abstract

**Aim:**

To ascertain the actual outcomes of oncofertility care in young women to provide more appropriate care.

**Materials & methods:**

We analyzed the data of 67 female patients under 43 years of age who underwent oncofertility care between January 2015 and September 2019.

**Results:**

There were 28 patients with breast cancer, 19 patients with hematologic cancer and 20 patients with other cancer diagnoses. Breast cancer patients tended to take longer than hematologic cancer patients to initiate oncofertility treatment. Despite undergoing oncofertility care, seven of nine pregnant patients did not choose assisted reproductive technology (ART).

**Conclusion:**

As spontaneous pregnancies were more common than ART pregnancies in our study, pregnancy by not only ART but also non-ART method is a viable option for young cancer survivors.

## Lay abstract

We conducted a study about oncofertility care and pregnancy outcomes in female cancer survivors under 43 years old. Even after chemotherapy, pregnancy by not only assisted reproductive technology (ART) but also non-ART method is a viable option for young cancer survivors. Social support as well as ART advancement is important for young cancer survivors and the low marriage rate may contribute to the low pregnancy rate.

Cancer treatments such as alkylating agents and pelvic irradiation expose patients to an increased risk of post-treatment infertility [1], which diminishes patients’ quality of life [2]. The future ability to have children has been cited as one of the top five unmet needs of adolescent cancer patients, along with health, work/school, romantic relationships and close friendships [3]. Consideration of post-treatment life should be an essential part of treatment planning for adolescent cancer patients, as advances in cancer therapy have dramatically increased the long-term survival rate of adolescent and young adult (AYA) cancer survivors aged 15–39 years. In particular, the survival rate of malignant lymphoma in childhood has risen to 80–90% [4,5]. Thus, the need for oncofertility treatment/care is increasing, yet the available data on marital relationships, conception methods and pregnancy outcomes among AYA cancer survivors remains insufficient [6]. It has been reported that, compared with women in the general population, female cancer survivors have lower marriage rates [7], lower pregnancy and birth rates and higher preterm delivery rates [6,8].

Oncofertility care for female cancer survivors can include embryo/oocyte cryopreservation, ovarian tissue cryopreservation and gonadotropin-releasing hormone agonist (GnRHa) therapy. The Japan Agency for Medical Research and Development reports that more than 1000 embryos or oocytes and more than 100 ovarian tissue samples were cryopreserved for cancer patients between January 2011 and December 2015 [9]. Among infertile patients, the pregnancy rate per cryopreserved embryo is 30–35% (according to the 2015 data from the Japan Society of Obstetrics and Gynecology [14]), while the pregnancy rate per cryopreserved oocyte is 4.5–12% [10]. Ovarian tissue auto-transplantation is also increasingly successful: the number of live births resulting from this method had exceeded 130 by June 2017 and has probably exceeded 200 in 2020 [11,12]. The interpretation of *in vitro* fertilization data remains controversial, yet, in a series of 60 patients treated with ovarian tissue auto-transplantation, the pregnancy rate was 50% and the live birth rate was 41% [12]. Ovarian tissue cryopreservation and auto-transplantation are performed around the world and are no longer considered entirely novel; in some countries, they are accepted as a non-experimental fertility preservation option for cancer patients. These options are also suitable for prepubescent females, as they neither delay cancer management nor involve hormonal stimulation [12].

Although these methods of preserving fertility among cancer survivors are becoming more typical and apparently more successful, there are few reports examining their actual outcomes such as marriage rates and conception methods after cancer treatment. Our hospital, one of the leading cancer care hospitals in Japan, has been providing oncofertility care to AYA cancer patients since January 2015, and currently operates as a central hub of the oncofertility network in the surrounding area. Since we routinely provide comprehensive follow-up regarding both cancer and reproductive care among AYA cancer patients, we have been able to accumulate a great deal of detailed information on reproduction after cancer treatment. To ascertain marriage rates and pregnancy methods and measure the success of oncofertility care in cancer survivors, we studied a series of cancer treatment cohorts and their pregnancy outcomes at our hospital.

## Materials & methods

### Design

This retrospective study was based on data collected between January 2015 and September 2019. We studied oncofertility care and pregnancy outcomes in female patients under the age of 43 years who were treated at our hospital. Patients were extracted from the medical records and oocyte pick up data of patients who visited our department by keywords such as oncofertility care and cancer. Oncofertility care included cryopreservation of oocytes and ovarian tissue as well as GnRHa therapy. The two largest patient cohorts receiving oncofertility care at our hospital were breast cancer patients and hematological cancer patients; we compared these cohorts through subanalyses of the following practices and outcomes: rates of cryopreservation of oocytes and ovarian tissue; mean number of egg retrieval cycles and mean number of egg retrievals and rates of marriage and pregnancy in patients over the age of 20 years.

Cryopreservation of oocytes and ovarian tissue was performed according to the vitrification method using the Cryotop and OvaCryoKit (Kitazato, Fuji, Japan); these procedures have been described elsewhere [13,14]. In addition, ovariectomy was performed by laparoscopic surgery on one side of the ovary in all cases, except in one individual who was undergoing laparotomy for a coexisting primary disease.

### Statistical analyses

The chi-square test was used to compare the proportions of patients who underwent cryopreservation of oocytes and ovarian tissue and the Kruskal–Wallis test was used to compare the mean number of oocytes preserved. A p-value <0.05 was considered significant.

## Results

**Figure 1.**
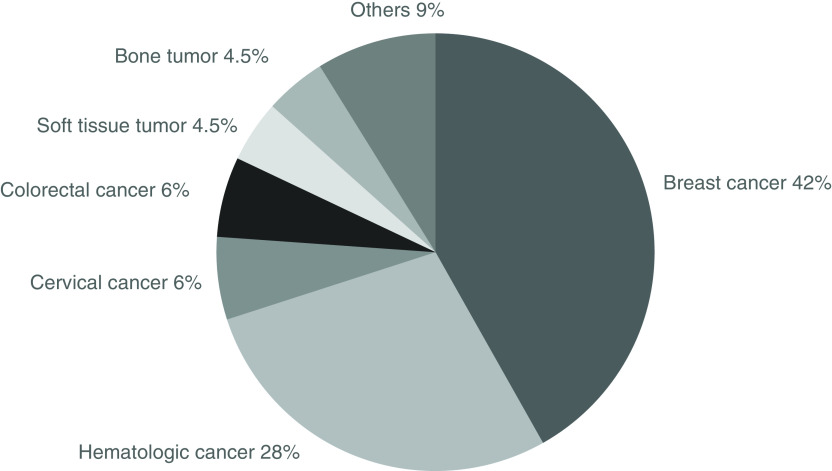
Type of cancer in 67 patients. The diagnosis was breast cancer in 28 (41.8%), hematologic cancer in 19 (28.4%), cervical cancer in 4 (6.0%), colon cancer in 4 (6.0%), soft tissue tumor in 3 (4.5%), bone tumor in 3 (4.5%) and other cancer in 6 (9.0%) (one diagnosis each of lung cancer, liver tumor, thyroid cancer, brain tumor, middle nasal passage tumor and tongue cancer).

A total of 67 female patients received oncofertility care at our hospital. They accounted for about 5.6% of cancer patients (n = 1200) under 43 years old who were treated at our hospital. The diagnosis was breast cancer in 28 (41.8%), hematologic cancer in 19 (28.4%), cervical cancer in four (6.0%), colon cancer in four (6.0%), soft tissue tumor in three (4.5%), bone tumor in three (4.5%) and other cancer in six (9.0%) (one diagnosis each of lung cancer, liver tumor, thyroid cancer, brain tumor, middle nasal passage tumor and tongue cancer) (Figure 1). These patients included three cases with unknown pregnancy and childbirth outcomes due to transfer to another hospital or interruption of hospital visits.

The mean age at first visit to our department was 30 ± 7.7 years old, 45 patients (67.2%) were referred to our department before starting treatment for primary disease and the mean number of days from referral to this department to starting treatment for primary disease was 22.2 ± 14.6 days. Cryopreservation of oocytes, cryopreservation of ovarian tissue and GnRHa therapy were performed in 45 cases (67.2%), eight cases (11.9%) and 43 cases (64.2%), respectively (Table 1). In 43 out of 53 cases (81.9%) of patients who received cryopreservation of oocytes and ovarian tissue also subsequently received GnRHa therapy for ovarian protection.

**Table 1.** Background of 67 patients in this study.

Mean age at first visit	30.0 ± 7.7 years
Timing of fertility preservation discussion	Before cancer treatment: 45 cases (67.2%) Average days until the start of cancer treatment: 22.2 ± 14.6 days
Cryopreservation of ovarian tissue	8 cases (11.9%)
Cryopreservation of oocyte	45 cases (67.2%)
GnRH agonist^†^	43 cases (64.2%)

† In 81.1% (43 out of 53 cases) of patients who underwent cryopreservation of oocytes and ovarian tissue, they also subsequently received GnRH agonist therapy for the purpose of ovarian protection.

GnRH: Gonadotropin-releasing hormone.

**Figure 2.**
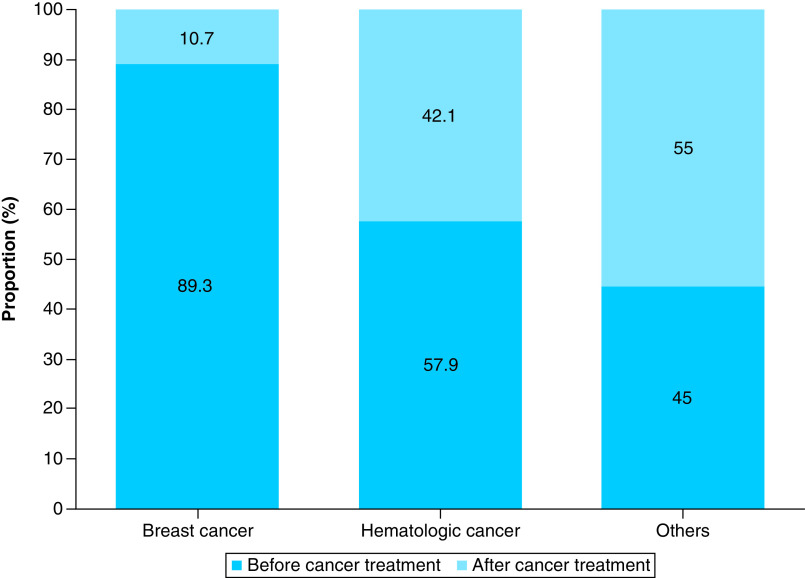
Timing of fertility preservation discussion for each type of cancer. We compared 28 cases of breast cancer, 19 cases of hematologic cancer, and 20 cases of other cancer as a sub-analysis. The proportion of each group of patients who visited our department before starting cancer treatment were 89.3, 57.9 and 45.0%.

Since the breast cancer patients and hematologic cancer patients accounted for a large proportion of all cancer patients, we compared the 28 breast cancer patients (the breast cancer group) with the 19 cases of hematologic cancer patients (the hematologic cancer group) and the 20 other cancer patients (the other cancer group) as a sub-analysis. The proportions of patients who visited our department before starting cancer treatment were 89.3% in the breast cancer group, 57.9% in the hematologic cancer group and 45.0% in the other cancer group (Figure 2). The mean durations between first visit to our department and starting treatment were 30.1 days in the breast cancer group, 6.3 days in the hematologic cancer group and 19.4 days in the other cancer group. The breast cancer group tended to have longer intervals between diagnosis and starting treatment than the hematologic cancer group.

**Figure 3.**
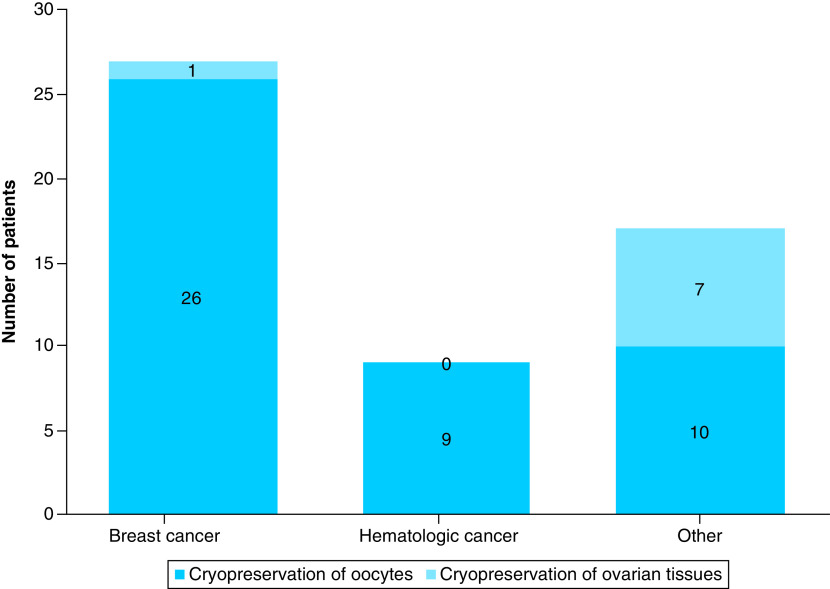
Proportion of cryopreservation of oocytes and ovarian tissues for each type of cancer. Cryopreservation of 26 oocytes and one ovarian tissue sample was conducted in 96.4% of breast cancer group (27 out of 28 cases). Cryopreservation of nine oocytes and no ovarian tissue sample was conducted in 47.4% of hematologic cancer group (nine out of 19 cases). And cryopreservation of ten oocytes and seven ovarian tissue samples was conducted in 85.0% of other cancer group (17 out of 20 cases). The rate of oocyte and ovarian tissue cryopreservation in the hematologic cancer group was lower than that in the breast cancer group and the other cancer group (p = 0.0002).

In the breast cancer group, 26 patients received oocyte cryopreservation and one received ovarian tissue cryopreservation for a total of 96.4% (27 out of 28 cases) participation in cryopreservation. In the hematologic cancer group, nine patients received oocyte cryopreservation and none received ovarian tissue cryopreservation for a total of 47.4% (nine out of 19 cases) participation in cryopreservation. In the other cancer group, ten patients received oocyte cryopreservation and seven cases received ovarian tissue cryopreservation for a total of 85.0% (17 out of 20 cases) participation in cryopreservation (Figure 3). The rates of oocyte and ovarian tissue cryopreservation were lower in the hematologic cancer group than in the breast cancer group or the other cancer group (p = 0.0002).

Table 2 presents details on the patients who received oocyte cryopreservation. The mean number of egg retrieval cycles and the mean number of cryopreserved oocytes were 1.8 ± 1.1 cycles and 15.0 ± 9.0 oocytes in the breast cancer group, 1.2 ± 0.6 cycles and 3.9 ± 2.8 in the hematologic cancer group and 1.1 ± 0.3 cycles and 14.2 ± 6.3 in the other cancer group. The numbers of patients whose oocytes were cryopreserved before chemotherapy were 24 (92.3%) in the breast cancer group, none in the hematologic cancer group and nine (45.0%) in the other cancer group. The mean number of egg retrieval cycles and the mean number of cryopreserved oocytes were both lower in the hematologic cancer group than in the breast cancer group or the other cancer group (p < 0.0001), probably because most of the hematologic cancer patients had started chemotherapy elsewhere before being referred to our outpatient clinic.

**Table 2.** Comparison of oocytes cryopreservation cases.

	Breast cancer	Hematologic cancer	Other cancer^†^
Mean cycles of egg retrieval	1.8 ± 1.1	1.2 ± 0.6	1.1 ± 0.3
Mean number of cryopreserved oocytes^‡^	15.0 ± 9.0	3.9 ± 2.8	14.2 ± 6.3
Number of patient for whom oocytes were cryopreserved before chemotherapy	24 cases (92.3%)	0 cases (0%)	9 cases (45.0%)
Pregnancy rate per cycle	12.5% One pregnancy per 8 cycles of embryo transfer	0% No transfer case	50.0% Two pregnancies per 4 cycles of embryo transfer

† ‘Other cancer’ includes three cervical cancer patients, three colon cancer patients, three soft tissue tumor patients, three bone tumor patients and five other cancer patients (one diagnosis each of lung cancer, liver tumor, thyroid cancer, brain tumor and middle nasal passage tumor).

‡ The mean number of cryopreserved oocytes in the hematologic cancer group was lower than in the breast cancer group and the other group (p < 0.0001).

Data on marriage rates and pregnancy permission by cancer treatment specialists in patients over the age of 20 are shown in Table 3. There were 28 (100%) patients over the age of 20 in the breast cancer group, 16 (84.2%) in the hematologic cancer group and 16 (80.0%) in the other cancer group. Of these, seven (25.0%) in the breast cancer group, four (25.0%) in the hematologic cancer group and four (25.0%) cases in the other cancer group were married. The marriage rate in the three cancer survivor groups combined was significantly lower than that among women of the same generation nationwide (p = 0.0015). Pregnancy permission by cancer treatment specialists was confirmed in only six cases (21.4%) in the breast cancer group, six cases (37.5%) in the hematologic cancer group and eight cases (50.0%) in the other cancer group.

**Table 3.** Marital and pregnancy permission status in patients aged 20 years and older.

	Breast cancer	Hematologic cancer	Other cancer^†^
Number of patients	28 cases (100%)	16 cases (84.2%)	16 cases (80.0%)
Married	7 cases (25.0%)	4 cases (25.0%)	4 cases (25.0%)
Pregnancy permission	6 cases (21.4%)	6 cases (37.5%)	8 cases (50.0%)

† ‘Other cancer’ includes four cervical cancer patients, four colon cancer patients, two soft tissue tumor patients, two bone tumor patients and four other cancer patients (one diagnosis each of lung cancer, thyroid cancer, middle nasal passage tumor and tongue cancer).

There were nine pregnancies as shown in Table 4. Two pregnancies were achieved through assisted reproductive technology (ART) while the remaining seven occurred through non-ART methods including spontaneous pregnancies. ART was attempted by only three patients: two breast cancer patients (five and three cycles of embryo transfer [ET] , respectively) and one other cancer patient (four cycles); the outcomes of these attempts were a sustained pregnancy in one of the two breast cancer patients and two pregnancies, both ending in early miscarriage, in the one other cancer patient. Regarding the non-ART pregnancies, spontaneous pregnancy and delivery occurred in three cases, pregnancy and delivery was achieved through a non-ART method in one case and three spontaneous pregnancies were ongoing at the time of this writing. There were four full-term deliveries, three of which were vaginal deliveries while the remaining one was a cesarean delivery due to fetal malpresentation. All of these pregnancies were free from complications and there were no cases in which ovarian tissue was thaw-transplanted.

**Table 4.** Pregnancy outcomes including nine pregnancies, two resulting from assisted reproductive technology and seven from non-assisted reproductive technology methods.

	Mean age at first visit (years)	Main indications	Cancer treatment	Time to pregnancy from end of cancer treatment	Marital status	Oncofertility care	Pregnancy outcome
1	30	Breast cancer	Surgery, hormone therapy	10 months	Married	Cryopreservation of embryos	Pregnancy due to timing method and successful delivery
2	32	Breast cancer	Surgery, chemotherapy	8 months	Married	Cryopreservation of oocytes	Spontaneous pregnancy and successful delivery
3	30	Breast cancer	Surgery, chemotherapy, radiation	52 months	Married	Cryopreservation of oocytes after chemotherapy	Current spontaneous pregnancy
4	35	Breast cancer	Surgery, radiation, hormone therapy	45 months	Married	Cryopreservation of embryos	Current pregnancy due to 3 cycles of embryo transfer
5	25	Hematologic cancer	Chemotherapy	57 months	Married	GnRH agonist	Current spontaneous pregnancy
6	34	Hematologic cancer	Chemotherapy	28 months	Married	GnRH agonist and cryopreservation of oocytes after chemotherapy	Spontaneous pregnancy and successful delivery
7	35	Cervical cancer	Surgery, chemotherapy	2 months	Married	Cryopreservation of embryos	Two pregnancies due to 4 cycles of embryo transfer but all miscarriages
8	23	Thyroid cancer	Surgery, chemotherapy, radiation	9 months	Married	Cryopreservation of oocytes	Spontaneous pregnancy and successful delivery
9	25	Chondrosarcoma	Surgery, chemotherapy	3 months	Married	GnRH agonist	Current spontaneous pregnancy

ART: Assisted reproductive technology; GnRH: Gonadotropin-releasing hormone.

## Discussion

The purpose of this study is to investigate the rates at which cancer survivors actually receive the available forms of oncofertility care and the outcomes of this care, and to determine what causes infertility in female cancer survivors who wish to become pregnant. Although our number of patients was small, our patient characteristics and outcomes were not much different from those in previous reports such as that by Sanada *et al.* [1]. In this study, we uncovered three findings worth noting. First, hematologic cancer patients did not usually receive fertility preserving treatments before starting primary cancer treatment, Second, since the marriage rate among cancer survivors is low, few patients actually received ART after cancer treatment. Finally, most of the pregnancies that did occur did not involve ART.

In this study, 67.2% of patients were informed of fertility preservation options before cancer treatment, indicating that these options are offered fairly consistently. A systematic literature review revealed 28 papers providing information on the optimal timing and methods for initiating fertility preservation. Another review found that the rate at which fertility counseling was offered prior to cancer treatment varied widely from 6 to 78% [15]. However, not all patients who desire oncofertility care are good candidates for oocyte and/or ovarian tissue cryopreservation and certain criteria should be established for when these methods should be offered. According to the Edinburgh Criteria, it is important to establish clear criteria that will ensure that oncofertility treatment is offered only in appropriate situations, in other words, when there is a reasonable likelihood of survival for more than 5 years after treatment and when the probability of premature ovarian insufficiency is greater than 50% [16]. Providing patients with clear information on appropriate options is a crucial aspect of oncofertility treatments.

Although there are pros and cons regarding the effects of GnRHa therapy [17,18], 64.2% of our patients received it. In cases where oocyte and/or ovarian tissue cryopreservation is not feasible, perhaps because of an insufficient interval between diagnosis and the start of treatment, GnRHa therapy should be offered as an option for protecting the ovaries. We have previously shown that GnRHa during chemotherapy is useful, especially for patients over the age of 30 [19].

In contrast to breast cancer patients, hematologic cancer patients did not have much time between arriving at our clinic and starting primary disease treatment, and the proportion of patients who received cryopreservation of oocytes and ovarian tissue was low. Furthermore, all of the hematologic cancer patients who did receive cryopreservation of oocytes received it after chemotherapy and their mean number of cryopreserved oocytes was lower than that among patients with other types of cancer. It is generally reported that a breast cancer prognosis is not adversely affected if adjuvant treatment such as chemotherapy is delayed for up to 12 weeks after surgery [20]. Likewise, another group has reported that the 5-year survival rate for breast cancer does not decrease unless treatment is delayed by 6 weeks or more from diagnosis [21]. While relatively long interval time before starting chemotherapy is therefore possible in breast cancer, acute leukemia must be treated as soon as possible. Consequently, there is not enough time to complete the egg retrieval process before beginning chemotherapy [22]. Fertilization and embryonic development rates are significantly reduced in mice treated with cyclophosphamide compared with a control group [22]. Furthermore, alkylating agents such as cyclophosphamide increase the risk of acute ovarian failure [23]. Thus, chemotherapy is likely to reduce the numbers of eggs that can be retrieved. Oncologists and patients must be aware of the risk of decreased egg counts, fertilization rates and embryonic development rates that is associated with chemotherapy and should consider early intervention for oncofertility care.

The marriage rate among our patients aged 20 years and older was about 25% in each cancer type group. Nationwide, in contrast, marriage rates are 36.3% among women 25–29 years old; 61.0% among women 30–34 years old and 69.8% among women 35–39 years old [24]. Therefore the marriage rate was lower among our patients than among women of the same generation nationwide. In addition, out of 32 patients who were 20 years of age or older with no partner at the time of their first visit to our department, only four got married during follow-up: two breast cancer patients, one hematologic cancer patient and one other cancer patient. This indicates that, among patients who are without partners when they start oncofertility care, both the marriage rate and the likelihood of attempting pregnancy are low compared with women in the general population. Previous reports have similarly indicated that young cancer survivors have a lower marriage rate than their healthy siblings [25]. One reason for the low marriage rate among young cancer survivors is unstable income. Additionally, they tend to experience fatigue, pain, anxiety, depression, recurrence and side effects of treatment for primary disease. Therefore, they are at high risk of job separation and low income [26]. Another reason is the fear of being rejected upon disclosing that they are cancer survivors, which may limit their pursuit of romantic relationships [27]. If these situations are not addressed, some cancer survivors will not able to achieve full quality of life with regard to pregnancy and childbirth even if oncofertility care improves in the future.

In terms of pregnancy outcomes, by the end of this study, 15 patients were married and nine had become pregnant. Seven of the nine pregnancies did not involve ART. The rate of pregnancy achieved through fertility awareness methods and intrauterine insemination among our patients was high compared with the rates previously reported for comparable patient groups (11.70–15.95%) [28]. This suggests that married cancer survivors can hope to conceive by methods other than ART even if they have cryopreserved oocytes. This is very interesting data, especially as it differs from that for most infertile patients. Only three patients, two in the breast cancer group and one in the other cancer group, attempted thawed ET using preserved embryos. One breast cancer patient had undergone five cycles of ET and had not become pregnant, while the other had undergone three cycles and was currently pregnant at the time of writing; the one other cancer patient had undergone four cycles of ET and become pregnant twice, but both pregnancies ended in early miscarriage. The success rate of conception through thawed ET is low because these patients had only one chance for egg retrieval prior to treatment and because most of these cases involved early embryo freezing. Given that the number of ART after chemotherapy is still small, however, we should perhaps not draw definitive conclusions until after additional cases have been accumulated.

Of the seven patients who conceived without ET, five had access to cryopreserved embryos or unfertilized oocytes but did not use them. This is in sharp contrast to the report by Alvarez and Ramanathan that only four of 531 female cancer patients aged 17–43 years experienced spontaneous pregnancy while another 22 conceived through ET [29]. Unlike previous studies, our study suggests that, even for patients whose oocytes and embryos are cryopreserved, we should consider not only ART but also spontaneous pregnancy as an option when considering how to achieve a balance with the primary disease.

The optimal timing of pregnancy after fertility preservation varies depending on the primary disease, and there are no fixed pregnancy permission standards informing the decision of when to conceive. In this study, pregnancy permission by cancer treatment specialists was expressed by six (21.4%) breast cancer patients, six (37.5%) hematologic cancer patients and eight (50.0%) other cancer patients, but we could not show clear trends pointing toward particular criteria for the appropriate timing of pregnancy. For breast cancer, the POSITIVE trial (JBCRG-23), which temporarily interrupts hormone therapy, permits pregnancy during that time and evaluates the risk of breast cancer recurrence, is currently being conducted and attempts to determine the appropriate timing for pregnancy are made. It is difficult to establish standards for pregnancy permission, but at the very least, cancer treatment specialists and reproductive medicine specialists should collaborate to develop an accurate understanding of each patient’s current situation, agree on mutual policies and consider all patient’s best interests.

Giving as many female cancer survivors as possible the option of conceiving will require, not only improvements in reproductive medicine but also stronger social support enabling better quality of life after cancer treatment. To that end, it is important that we continue to perform long-term follow-up even after fertility preservation is complete and that we strive to provide reproductive medical care according to each patient’s situation.

## Conclusion

The low rates of both marriage and fertility treatments such as ART among AYA cancer survivors demonstrate that we need to improve not only reproductive medicine but also social support in order to increase pregnancy rates among AYA cancer survivors. Additionally, it is important for patients and their reproductive specialists to consider the most appropriate conception method for each case and to recall that spontaneous pregnancy can also be expected after chemotherapy. Further study, including both ongoing observation of our present patients and the accumulation of more cases, is needed in order to confirm our findings.

## Future perspective

As the time that can be spent on oncofertility care before starting treatment for the primary disease and the content of oncofertility care differ depending on the primary disease, oncologist and reproductive medicine specialist should work together from early on. We should consider the lives of AYA cancer survivors after cancer treatment and the most appropriate pregnancy method rather than merely utilizing ART.

**Summary points**

**Introduction**

- Cancer treatments such as alkylating agents and pelvic irradiation expose patients to an increased risk of post-treatment infertility.
- As the long-term survival rate of cancer patients increases, the need for oncofertility treatment/care increases among adolescent and young adult cancer survivors.
- Embryo cryopreservation, oocyte cryopreservation, ovarian tissue cryopreservation, and gonadotropin-releasing hormone agonist therapy are methods of female oncofertility care.

**Result of retrospective study**

- This study included the data of 67 female patients under 43 years of age who underwent oncofertility care between January 2015 and September 2019.
- There were 28 patients with breast cancer (41.8%), 19 patients with hematologic cancer (28.4%) and 20 patients with other cancer diagnoses.
- In contrast to the breast cancer group, the hematologic cancer group did not have much time before starting primary disease treatment and the ratio of patients who underwent cryopreservation of oocytes was low because the hematologic cancer group had insufficient time for egg retrieval.
- The marriage rate in patients aged 20 years and older was about 25% in each group; this was lower than that of women of the same generation nationwide.
- There were only four out of 32 patients who were 20 years of age or older with no partner at the time of their first visit to our department who got married during follow-up.
- A total of 15 patients were married and nine became pregnant in this study, notably seven of the nine pregnancies involve non-assisted reproductive technology. Only three patients actually received assisted reproductive technology after cancer treatment.

## References

[1] Sanada Y, Harada M, Kunitomi C A Japanese nationwide survey on the cryopreservation of embryos, oocytes and ovarian tissue for cancer patients. J. Obstet. Gynaecol. Res. 45(10), 2021–2028 (2019). 31364239 10.1111/jog.14073

[2] Tarasiewicz M, Martynowicz I, Knapp P, Sieczynski P. “Oncofertility” procedures in children and adolescents. Pediatr. Endocrinol. Diabetes Metab. 25(3), 144–149 (2019). 31769272 10.5114/pedm.2019.87710

[3] Klosky JL, Simmons JL, Russell KM Fertility as a priority among at-risk adolescent males newly diagnosed with cancer and their parents. Support. Care Cancer 23(2), 333–341 (2015). 25082365 10.1007/s00520-014-2366-1 PMC4289648

[4] Mauz-Korholz C, Metzger ML, Kelly KM Pediatric Hodgkin Lymphoma. J. Clin. Oncol. 33(27), 2975–2985 (2015). 26304892 10.1200/JCO.2014.59.4853

[5] Minard-Colin V, Brugieres L, Reiter A Non-Hodgkin lymphoma in children and adolescents: progress through effective collaboration, current knowledge, and challenges ahead. J. Clin. Oncol. 33(27), 2963–2974 (2015). 26304908 10.1200/JCO.2014.59.5827 PMC4979194

[6] Anderson RA, Brewster DH, Wood R The impact of cancer on subsequent chance of pregnancy: a population-based analysis. Hum. Reprod. 33(7), 1281–1290 (2018). 29912328 10.1093/humrep/dey216 PMC6012597

[7] Kirchhoff AC, Yi J, Wright J, Warner EL, Smith KR. Marriage and divorce among young adult cancer survivors. J. Cancer Surviv. 6(4), 441–450 (2012). 22956304 10.1007/s11764-012-0238-6 PMC6159935

[8] Van Dorp W, Haupt R, Anderson RA Reproductive function and outcomes in female survivors of childhood, adolescent, and young adult cancer: a review. J. Clin. Oncol. 36(21), 2169–2180 (2018). 29874135 10.1200/JCO.2017.76.3441 PMC7098836

[9] Harada M, Osuga Y. Fertility preservation for female cancer patients. Int. J. Clin. Oncol. 24(1), 28–33 (2019). 29502284 10.1007/s10147-018-1252-0

[10] Practice Committees of the American Society for Reproductive M. The Society for Assisted Reproductive T. Mature oocyte cryopreservation: a guideline. Fertil. Steril. 99(1), 37–43 (2013). 23083924 10.1016/j.fertnstert.2012.09.028

[11] Donnez J, Dolmans MM. Fertility preservation in women. N. Engl. J. Med. 377(17), 1657–1665 (2017). 29069558 10.1056/NEJMra1614676

[12] Dolmans MM, Falcone T, Patrizio P. Importance of patient selection to analyze *in vitro* fertilization outcome with transplanted cryopreserved ovarian tissue. Fertil. Steril. 114(2), 279–280 (2020). 32741467 10.1016/j.fertnstert.2020.04.050

[13] Kuwayama M, Vajta G, Kato O, Leibo SP. Highly efficient vitrification method for cryopreservation of human oocytes. Reprod. Biomed. Online 11(3), 300–308 (2005). 16176668 10.1016/s1472-6483(10)60837-1

[14] Suzuki N, Yoshioka N, Takae S Successful fertility preservation following ovarian tissue vitrification in patients with primary ovarian insufficiency. Hum. Reprod. 30(3), 608–615 (2015). 25567618 10.1093/humrep/deu353

[15] Anazodo A, Laws P, Logan S How can we improve oncofertility care for patients? A systematic scoping review of current international practice and models of care. Hum. Reprod. Update 25(2), 159–179 (2019). 30462263 10.1093/humupd/dmy038 PMC6390168

[16] Wallace WH, Smith AG, Kelsey TW, Edgar AE, Anderson RA. Fertility preservation for girls and young women with cancer: population-based validation of criteria for ovarian tissue cryopreservation. Lancet Oncol. 15(10), 1129–1136 (2014). 25130994 10.1016/S1470-2045(14)70334-1 PMC4153375

[17] Moore HC, Unger JM, Phillips KA Goserelin for ovarian protection during breast-cancer adjuvant chemotherapy. N. Engl. J. Med. 372(10), 923–932 (2015). 25738668 10.1056/NEJMoa1413204 PMC4405231

[18] Demeestere I, Brice P, Peccatori FA No evidence for the benefit of gonadotropin-releasing hormone agonist in preserving ovarian function and fertility in lymphoma survivors treated with chemotherapy: final long-term report of a prospective randomized trial. J. Clin. Oncol. 34(22), 2568–2574 (2016). 27217453 10.1200/JCO.2015.65.8864

[19] Kentaro I, Miyuki I, Sawako F, Hirohiko T, Akihito H, Masaki M. Gonadotropin-releasing hormone agonists reduced chemotherapy induced premature ovarian failure in patients over 30 years of age. J. Fertil. Preservation 3(1), 42–47 (2020).

[20] Lohrisch C, Paltiel C, Gelmon K Impact on survival of time from definitive surgery to initiation of adjuvant chemotherapy for early-stage breast cancer. J. Clin. Oncol. 24(30), 4888–4894 (2006). 17015884 10.1200/JCO.2005.01.6089

[21] Smith EC, Ziogas A, Anton-Culver H. Delay in surgical treatment and survival after breast cancer diagnosis in young women by race/ethnicity. JAMA Surg. 148(6), 516–523 (2013). 23615681 10.1001/jamasurg.2013.1680

[22] Takai Y. Recent advances in oncofertility care worldwide and in Japan. Reprod. Med. Biol. 17(4), 356–368 (2018). 30377391 10.1002/rmb2.12214 PMC6194250

[23] Chemaitilly W, Mertens AC, Mitby P Acute ovarian failure in the childhood cancer survivor study. J. Clin. Endocrinol. Metab. 91(5), 1723–1728 (2006). 16492690 10.1210/jc.2006-0020

[24] Statistics Bureau, Ministry of Internal Affairs and Communications. National Census Results. (2015). https://www.stat.go.jp/data/kokusei/2015/kekka/kihon1/pdf/gaiyou1.pdf

[25] Brinkman TM, Recklitis CJ, Michel G, Grootenhuis MA, Klosky JL. Psychological symptoms, social outcomes, socioeconomic attainment, and health behaviors among survivors of childhood cancer: current state of the literature. J. Clin. Oncol. 36(21), 2190–2197 (2018). 29874134 10.1200/JCO.2017.76.5552 PMC6053297

[26] Sheppard DM, Frost D, Jefford M, O'connor M, Halkett G. ‘Beyond Cancer’: a study protocol of a multimodal occupational rehabilitation programme to support breast cancer survivors to return work. BMJ Open 9(12), e032505 (2019). 10.1136/bmjopen-2019-032505 PMC6924857 31843840

[27] Murphy D, Klosky JL, Reed DR, Termuhlen AM, Shannon SV, Quinn GP. The importance of assessing priorities of reproductive health concerns among adolescent and young adult patients with cancer. Cancer 121(15), 2529–2536 (2015). 26054052 10.1002/cncr.29466

[28] Ye F, Cao W, Lin J The pregnancy outcomes of intrauterine insemination with husband's sperm in natural cycles versus ovulation stimulated cycles: a retrospective study. Biosci. Trends 12(5), 463–469 (2018). 30473553 10.5582/bst.2018.01164

[29] Alvarez RM, Ramanathan P. Fertility preservation in female oncology patients: the influence of the type of cancer on ovarian stimulation response. Hum. Reprod. 33(11), 2051–2059 (2018). 27370358 10.1093/humrep/dew158

